# Reduced reward‐related neural response to mimicry in individuals with autism

**DOI:** 10.1111/ejn.13620

**Published:** 2017-07-12

**Authors:** Chun‐Ting Hsu, Janina Neufeld, Bhismadev Chakrabarti

**Affiliations:** ^1^ Centre for Integrative Neuroscience and Neurodynamics School of Psychology and Clinical Language Sciences University of Reading Whiteknights Reading RG6 6AL UK; ^2^ Center of Neurodevelopmental Disorders at Karolinska Institutet (KIND) Karolinska Institutet Stockholm Sweden; ^3^ Present address: Brain, Language, and Computation Lab Department of Psychology Pennsylvania State University University Park PA 16802 USA

**Keywords:** autism, fMRI, mimicry, reward, social

## Abstract

Mimicry is a facilitator of social bonds in humans, from infancy. This facilitation is made possible through changing the reward value of social stimuli; for example, we like and affiliate more with people who mimic us. Autism spectrum disorders (ASD) are marked by difficulties in forming social bonds. In this study, we investigate whether the reward‐related neural response to being mimicked is altered in individuals with ASD, using a simple conditioning paradigm. Multiple studies in humans and nonhuman primates have established a crucial role for the ventral striatal (VS) region in responding to rewards. In this study, adults with ASD and matched controls first underwent a conditioning task outside the scanner, where they were mimicked by one face and ‘anti‐mimicked’ by another. In the second part, participants passively viewed the conditioned faces in a 3T MRI scanner using a multi‐echo sequence. The differential neural response towards mimicking vs. anti‐mimicking faces in the VS was tested for group differences as well as an association with self‐reported autistic traits. Multiple regression analysis revealed lower left VS response to mimicry (mimicking > anti‐mimicking faces) in the ASD group compared to controls. The VS response to mimicry was negatively correlated with autistic traits across the whole sample. Our results suggest that for individuals with ASD and high autistic traits, being mimicked is associated with lower reward‐related neural response. This result points to a potential mechanism underlying the difficulties reported by many of individuals with ASD in building social rapport.

## Introduction

Mimicry is a fundamental feature of human social behaviour and often leads to increased liking and perceived closeness towards the mimicker and facilitates prosocial behaviour (van Baaren *et al*., 2003, 2004; Ashton–James *et al*., 2007; Stel & Vonk, 2010). It has been suggested to function as ‘social glue’, a key mechanism that helps to build social rapport (Lakin *et al*., 2003; Chartrand & Lakin, 2013). It is believed to play this vital role from early on in human development, where face‐to‐face mimicry between the caregiver and infant helps in building social bonds (Trevarthen, 1998). Mimicry‐related processes lead to greater preferential attention and positive responses, both in macaque as well as human infants (Meltzoff & Brooks, 2001; Sclafani *et al*., 2015). In adult humans, increased activity in brain regions involved in reward processing has been observed when participants watched others being mimicked (Kühn *et al*., 2010). These findings suggest that mimicry is associated with a reward‐related response.

To systematically test the effect of mimicry on reward value of social stimuli, we recently conducted an eye‐tracking study in neurotypical adults. In this experiment, participants were first conditioned through trials where they were mimicked by certain faces and ‘anti‐mimicked’ (i.e. made an expression different from that of the participant) by other faces. This conditioning phase was followed by a test phase where mimicking and anti‐mimicking faces were presented side by side using a preferential looking paradigm. This experiment revealed that participants looked longer at faces that mimicked them compared to those that did not (Neufeld & Chakrabarti, 2016). The strength of this preferential gaze bias for mimicking faces correlated positively with trait empathy, suggesting that more empathic individuals were more sensitive to the manipulation. This finding provides further support for the role of mimicry in changing the reward value of social stimuli and is in line with the suggestion that mimicry is an important learning signal from the parent contributing to the development of empathy (Meltzoff, 2007). Atypical reward response to mimicry could therefore lead to impairments in social cognition and empathy, as seen in individuals with ASD.

Children with ASD often respond abnormally to social stimuli, such as social sounds or faces (Dawson, 2008). According to the social motivation theory in autism, those with ASD are less motivated to attend to social stimuli because they do not experience them as rewarding (Chevallier *et al*., 2012). It has been suggested that a lack of attention to social stimuli and atypicalities in spontaneous facial mimicry reinforce each other, leading to further deficits in social cognition seen in ASD (Dawson, 2008; Hamilton, 2015). In the light of the putative role of mimicry in the development of empathy (Meltzoff & Decety, 2003), an atypical link between mimicry and reward in ASD might be one of the roots of the deficits in empathy in these individuals. While evidence for a domain‐general impairment in mimicry in ASD is weak (Hamilton, 2013), paradigms measuring specific aspects of mimicry, such as spontaneous facial mimicry, often demonstrate atypicalities in individuals with high autistic traits and those with a clinical diagnosis of ASD (Beall *et al*., 2008; Oberman *et al*., 2009). Studying this link systematically in a clinical sample compared to neurotypicals can therefore lead to useful insights into the underlying mechanisms.

The link between reward and mimicry can be examined from at least two different approaches: first, the link between reward and the act of mimicking, and second, the link between reward and the act of being mimicked. Using the first approach, recent EMG, fMRI and EEG studies have investigated this link and demonstrated that increasing the reward value of faces leads to greater spontaneous facial mimicry (Sims *et al*., 2012), greater functional coupling between reward‐ and mimicry‐related brain areas (Sims *et al*., 2014) and greater mu suppression, considered to be an index of cortical motor simulation (Trilla‐Gros *et al*., 2015). The reward‐driven modulation of both spontaneous facial mimicry and the functional connectivity between ventral striatum (VS) and inferior frontal gyrus (IFG) were inversely correlated to autistic traits as measured by autism quotient (AQ), indicating that individuals high in autistic traits have a weaker link between reward and mimicry.

In contrast, there is a paucity of similar studies using the second approach in relation to ASD. In the present study, we addressed this gap in knowledge by testing the impact of being mimicked on reward‐related neural activity in adults with and without ASD. To test the reward‐related response to faces associated with mimicry (vs. anti‐mimicry), we focused specifically on the response of the VS. In humans, the term ‘ventral striatum’ refers to the nucleus accumbens and ventral aspects of the caudate and putamen. This region receives cortical input from the orbital frontal cortex and anterior cingulate cortex, as well as mesolimbic dopaminergic afferents. The VS projects to the ventral pallidum and to the ventral tegmental area and substantia nigra, which, in turn, project back to the prefrontal cortex, via the medial dorsal nucleus of the thalamus. This circuit is an integral part of the cortico‐basal ganglia system and plays a central role in reward processing in humans and other mammals. Previous studies have demonstrated that activity in VS is associated with anticipation of both primary and secondary rewards (Schultz *et al*., 2000; O'Doherty *et al*., 2002; O'Doherty, 2004; Haber & Knutson, 2010; Liu *et al*., 2011). Recent meta‐analyses of human neuroimaging studies further confirm the observations from electrophysiological studies in primates, in supporting the role of the VS in both anticipation and consummation of reward (Liu *et al*., 2011; Diekhof *et al*., 2012).

In line with the literature discussed above and our recent observation in neurotypicals using a preferential looking paradigm (Neufeld & Chakrabarti, 2016), we hypothesized that the rewarding effect of being mimicked by a face will be lower for individuals with ASD. Consequently, ASD individuals will demonstrate a reduced VS response to being mimicked, when compared to a matched group of neurotypical controls. To test this hypothesis, we conducted a mimicry conditioning experiment with a conditioning phase identical to that used by Neufeld & Chakrabarti (2016). During the test phase inside the MRI scanner, participants were presented with the faces that previously mimicked vs. those that did not. The differential response to mimicking vs. anti‐mimicking faces in the VS was then compared between groups and tested for a relationship with autistic traits.

## Materials and methods

Participants first underwent a conditioning phase outside the scanner during which they were mimicked or anti‐mimicked by different actors on screen (see section on Design and Procedure‐ conditioning phase). Facial EMG recorded during this phase to ensure that the participants were performing the correct facial expression. Subsequently, they were placed in a 3T Siemens Trio fMRI scanner where they completed the test phase. Participants also rated all face stimuli on a seven‐point Likert scale for likeability (1 = not likeable at all, 7 = very likeable) and attractiveness (1 = not attractive at all, 7 = very attractive).

### Participants

Thirty‐six adults clinically diagnosed with ASD using DSM‐IV criteria and 35 adults without any self‐reported neurological or psychiatric disorders (Table 1) were recruited from a database of research volunteers with and without ASD from in and around the University of Reading and received either a small compensation or credit points for their participation. All participants completed a nonverbal IQ test (Raven's matrices). All ASD participants had a confirmed ASD diagnosis from a registered clinic and were additionally assessed with the Autism Diagnostic Observation Schedule (ADOS) Module 4 (consensus of two researchers certified for reliability). All participants had normal or corrected‐to‐normal vision. Ethical approval for the study was obtained from the University Research Ethics Committee of the University of Reading, UK, and all participants provided informed consent. The study conforms to the norms laid out in the Declaration of Helsinki.

**Table 1:** Descriptive characteristics of all individuals whose data were included in the final analysis (NT = neurotypical and ASD = autism spectrum disorders)

Measure	NT (*n* = 30)		ASD (*n* = 26)		Statistical test	*P*‐value
	Mean (SD)	Range	Mean (SD)	Range		
Age	30.73 (2.09)	18–57	35.08 (2.24)	18–60	*t*‐test	0.16
Gender (M:F)	(18 : 12)	–	(15 : 11)	–	Chi‐square	0.86
Handedness (R:L:Ambi)	(24 : 6 : 0)	–	(19 : 6 : 1)	–	Chi‐square	0.43
IQ (Ravens Percentile)	48.5 (4.65)	6–90	55.5 (5.00)	2–96	*t*‐test	0.31
AQ	15.34 (4.98)	6–25	36.23 (7.66)	22–49	*t*‐test	< 0.0001
EQ	41.59 (9.64)	14–60	22.04 (9.55)	6–46	*t*‐test	< 0.0001

Ten ASD and five neurotypical participants were excluded in all. Reasons for exclusion were due to the current usage of anti‐psychotics (one ASD participant), the inability to finish the experiment (four ASD participants), poor test phase performance (two ASD and one neurotypical participants), being outside the age range (one ASD participant), structural anomaly (one neurotypical participant) and fMRI data quality issues (two ASD and three neurotypical participants). Nine of 26 ASD participants (six females) had ADOS total scores below the cut‐off for ASD of seven. However, failure to reach cut‐off scores on the ADOS *per se* did not lead to exclusion of ASD participants. The age, gender, handedness and IQ (Ravens percentile) of the remaining participants were matched between two groups and summarized in Table 1.

### Stimuli

Stimuli were derived from the Amsterdam Dynamic Facial Expression Set (ADFES) database (http://bit.ly/1dMyC2V). Stimuli for the conditioning phase consisted of 3‐s video clips of faces of four different actors (two female and two male faces). There were two videos per face: one showing a happy and one a sad expression. Each face had a neutral expression in the beginning which turned into a happy or sad expression after ~ 1 s and was kept until the end of the video. In the test phase, static images of the same faces with 80% neutral facial expressions were presented one at a time in front of a black screen. The static images for the test phase were created with Sqirlz Morph 2.1 (http://www.xiberpix.net/SqirlzMorph.html), morphing a neutral face with a happy one to prevent neutral faces to be perceived as threatening (Yoon & Zinbarg, 2008).

### Design and procedure – conditioning phase

**Figure 1:**
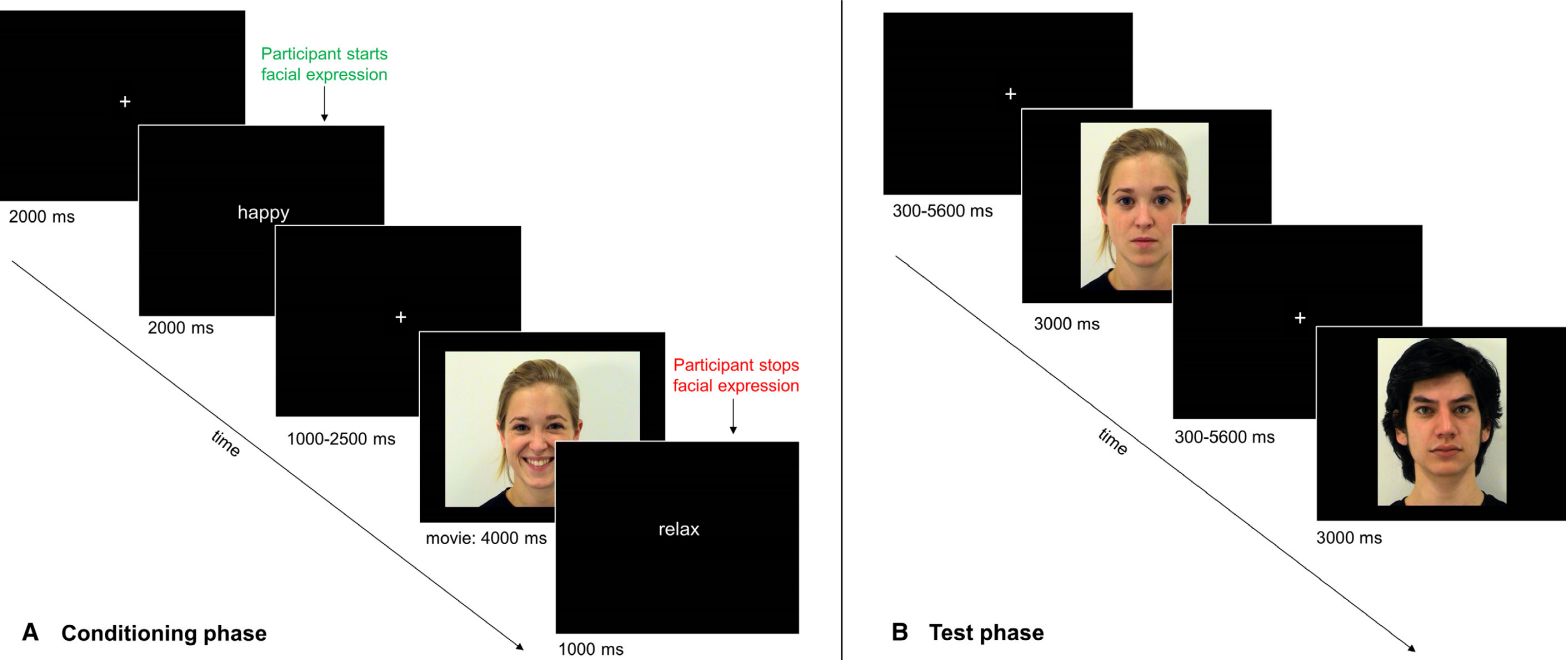
Schematic overview over the experimental procedure. **(**A) During the conditioning phase, participants were first instructed which expression (happy or sad) to perform in each trial. After a variable delay, a video appeared that displayed either the same (mimicking face) or the other expression (anti‐mimicking face). (B) During the test phase within the scanner, the same faces shown during the conditioning were presented one at a time as neutral still pictures while participants performed an oddball task to ensure their attention to the screen. They were asked to press a button when they saw a face that they had not seen earlier.

Stimuli were displayed using E‐Prime 2.2 (Psychology Software Tools, PA, USA) on a Viewsonic VE510s monitor (colour TFT active matrix XGA LCD 30.5 cm × 23 cm). The conditioning phase closely resembled that reported in Neufeld & Chakrabarti (2016). During the conditioning phase, participants were instructed to perform facial expressions (happy or sad) while they watched short clips of four different faces making happy or sad expressions (Fig. 1). To create the subjective experience of being mimicked (or anti‐mimicked) by the videos, it was crucial that the participants made the correct facial expression before they saw the face in the video performing the expression. To make sure that participants performed the correct expression in time, facial electromyographic (EMG) responses were measured during the mimicry conditioning phase using sensors placed over the Zygomaticus Major and Corrugator Supercilii. Electrode placement and hardware settings were identical to Sims *et al*. (2012) and Neufeld & Chakrabarti (2016). The signal was checked for each trial by visual inspection. Participants had to achieve a clearly visible signal increase in the correct muscle (Zygomaticus Major activity after the instruction ‘happy’ and Corrugator Supercilii after the instruction ‘sad’), before the onset of the facial expression in the video. If this was not the case, the trial was counted as error. The percentage of correctly performed expressions served as an inclusion criterion (> 70%) and as covariate in the analysis of BOLD response and rating outcomes. Prior to the experiment, participants read the task instructions and practised their happy and sad expression using a small mirror. After EMG electrode placement, participants completed a short practice session consisting of eight trials (two for each face, one with happy and one with sad expression). Participants were asked to make a happy expression as soon as they saw the word ‘happy’ and a sad expression as soon as they saw the word ‘sad’ on screen, and keep each expression until the word ‘relax’ was displayed. They were instructed to keep a relaxed face when seeing the word ‘neutral’. Between the expression cue and the word ‘relax’, a video of a face making a sad or happy expression was displayed for 4‐s after a variable delay (mean of 650 ms). This expression was either congruent or incongruent to the participant's expression. In neutral trials, participants kept a neutral face while simply watching the face making a happy or sad expression (neutral trials). Two of the four faces (one male and one female face) were associated with 90% congruent (mimicking faces) and two different faces (one male and one female) with 90% incongruent trials (anti‐mimicking faces). The remaining 10% of trials of each face identity were associated with neutral instructions to prevent participants from easily guessing the underlying conditioning procedure. (This awareness was assessed through a questionnaire at the end of the test phase and revealed that seven ASD and 12 neurotypical participants had correctly guessed the pattern in the conditioning task.)

For each face in the conditioning phase, half of the trials consisted of it making a happy expression, while the other half consisted of it making a sad expression. There were 20 conditioning trials per face (10 happy, 10 sad), resulting in 80 conditioning trials in total. After 40 trials, participants had a chance to take a break. Each half of the conditioning phase contained the same number of congruent, incongruent and neutral trials and the same number of happy and sad video presentations. Within each half, the stimulus order was randomized.

### Design and procedure – test phase

The test phase took place directly after the conditioning phase (with a 15–25 min delay needed for set‐up) and consisted of an oddball task, during which images of the same four identities used for conditioning were presented with a neutral facial expression (Fig. 1). One face presented at a time for 3‐seconds. Every stimulus was preceded by a fixation cross (white on black background), the duration of which was jittered ranging from 300 to 5600 ms. The duration of jitter and the order of presentation of stimuli were designed to maximize power for estimating the contrast of interest using OptSeq (http://www.surfer.nmr.mgh.harvard.edu/optseq). Two optimized sequences were created (order 1: mean ISI = 1989.77 ms, SD = 1046.26 ms; order 2: mean ISI = 1986.36 ms, SD = 1247.36 ms), and approximately half of the participants of each group were presented with one order and the other half with the other order. Each face was presented 20 times, resulting in 40 trials per condition (Panasiti *et al*., 2016). Eight ‘novel’ neutral faces (= oddball stimuli) were distributed among the target faces and participants were instructed to press a button on a device they held in their right hand when detecting a novel face to ensure their attention to the screen.

### Trait measurements

All participants but one in the neurotypical group completed the autism spectrum quotient (AQ, Baron‐Cohen *et al*., 2001) and the empathy quotient (EQ, Baron‐Cohen & Wheelwright, 2004) online (see Table 1 for summary AQ and EQ scores for both groups).

### Behavioural data analysis

Rating data from three neurotypical participants were lost due to technical errors. The likeability ratings for mimicking vs. non‐mimicking faces before and after conditioning were used in the repeated‐measure anova with mimicry and conditioning as within‐subject factors, and ASD diagnosis as a between‐subject factor. Handedness and conditioning accuracy were added as covariates.

### Regions of interest

**Figure 2:**
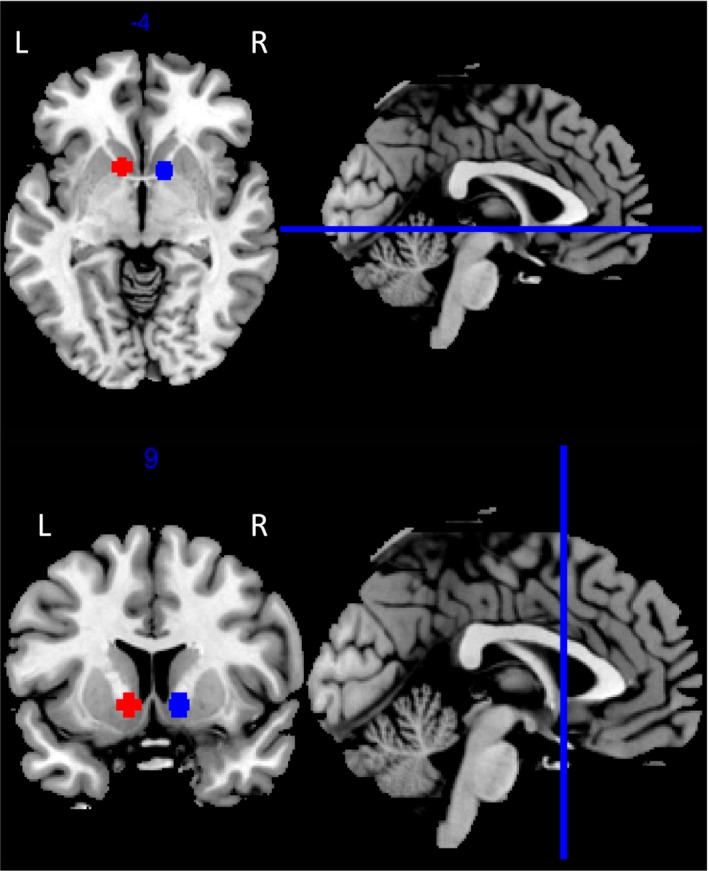
Predefined regions of interest in the left (red) and right (blue) VS based on a meta‐analysis of 142 neuroimaging studies of reward processing (Liu *et al*., 2011).

Regions of interest (ROIs) within the left and right VS were identified using coordinates reported in a meta‐analysis of neuroimaging studies of reward by Liu *et al*. (2011) [right (12, 8, −4); left (−10, 10, −4)]. The WFU PickAtlas tool (Maldjian *et al*., 2003) was used to draw spheres with 5 mm radius around the centre coordinates of the selected ROIs (Fig. 2).

### fMRI data acquisition and pre‐processing

Participants were scanned in a 3T Siemens TIM Trio MRI scanner with 32 channel head coil; 32 3‐mm‐thick axial slices were acquired in descending sequential order using a multi‐echo sequence, with three different echo times [TR = 2400 ms; TE (1; 2; 3) = 20; 36; 52 ms]. Multi‐echo sequences have been shown to have considerably greater signal to noise ratio for echo‐planar images (Lombardo *et al*., 2016). DICOM files were converted to NIfTI data image files using dcm2nii in MRICron. Pre‐processing and multi‐echo ICA (Kundu *et al*., 2012, 2013) were performed in AFNI (Cox, 1996). The first four volumes were discarded to allow for the stabilization of the magnetization. Procedures consisted of slice‐timing correction, realignment of the functional images for motion correction, and the functional to structural co‐registration. The multi‐echo‐ICA was then performed, and the BOLD (linear TE‐dependent signal decay) and non‐BOLD components were separated. The non‐BOLD components were used as nuisance regressors to de‐noise the functional data. The de‐noised functional images were converted to SPM 3D images with dcm2nii and spatially smoothed with a Gaussian kernel of FWHM 5 mm using SPM8 (http://www.fil.ion.ucl.ac.uk/spm).

### fMRI data analysis

Statistical parametric maps were calculated with multiple regressions of the data onto a model of the hemodynamic response (Friston *et al*., 1995). The first‐level general linear model analyses contained three regressors for mimicking, anti‐mimicking and oddball conditions, and each stimulus lasted 3‐s. Regressors were convolved with the canonical hemodynamic response function. For each ROI, the mean t‐statistics of the contrast (mimicking > anti‐mimicking faces) for each participant were extracted with MarsBaR (version 0.44) and used as dependent variables for the group‐level analysis. To test both categorical and dimensional approaches, two models of ordinary least squares regression including the handedness and conditioning accuracy as covariates were created. The first model tested the effect of group (neurotypical vs. ASD), while the second model tested the effect of AQ or EQ. Mean ± 3SD was used as the criteria to filter outliers, and none were identified. Two similar analyses were conducted at the whole brain level, (i) a random effect flexible factorial analysis with two factors: Group (ASD vs. Neurotypical) × Condition (mimicry vs. anti‐mimicry), and (ii) a random effect multiple regression with either AQ or EQ as the regressor. As in the ROI analysis, handedness and conditioning accuracy were entered in both models as covariates. Main effects of Group, Condition, and the interaction effect in the factorial analysis, as well as the effect of AQ in the multiple regression analysis, were checked. We imposed an initial voxel‐level threshold of uncorrected *P* < 0.001, and then a cluster‐level threshold of family‐wise error (FWE)‐corrected *P* < 0.05 for the entire image volume. The anatomical labels reported in the results were taken from the Talairach Daemon database (Lancaster *et al*., 1997, 2000) or the AAL atlas (Tzourio‐Mazoyer *et al*., 2002) incorporated in the WFU PickAtlas Tool (Maldjian *et al*., 2003). The Brodmann's areas (BA) were further checked with the Talairach Client using nearest grey matter search after coordinate transformation with the WFU PickAtlas Tool.

## Results

### Behavioural task results

Likeability ratings showed no significant main effect of conditioning (*F*_1,49_ = 1.70, *P* = 0.198), mimicry (*F*_1,49_ = 0.68, *P* = 0.414), group (*F*_1,49_ = 1.46, *P* = 0.232), and no significant interaction between conditioning, mimicry and group factors. Planned *post hoc* analysis in the control group showed a significant interaction between conditioning and mimicry (*F*_1,24_ = 5.29, *P* = 0.03), replicating previously published results (Neufeld & Chakrabarti, 2016).

Performance during the conditioning task (as assessed based on the EMG signal in the congruent muscle) did not differ between groups (96.8 ± 4.9% accuracy in controls, 94.9 ± 7.4% accuracy in ASD individuals).

### fMRI task results

**Figure 3:**
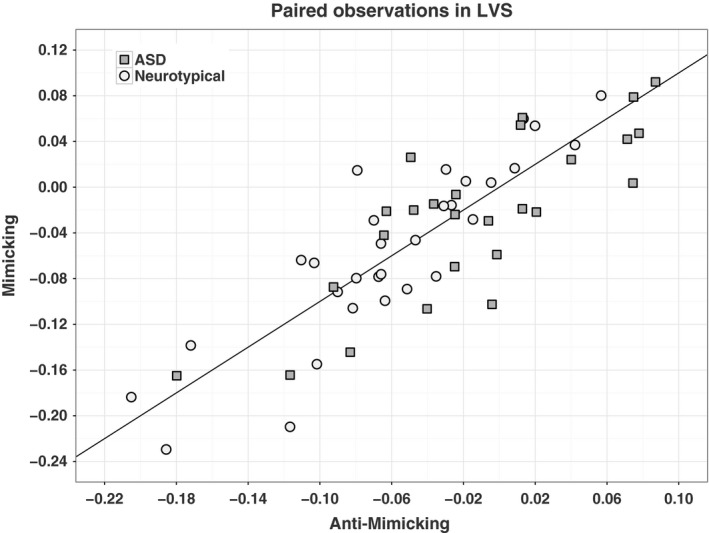
Left ventral striatal response to mimicking vs. anti‐mimicking faces in ASD and Neurotypical groups. Each point on this plot denotes an individual left VS response to mimicking and anti‐mimicking faces. Response to mimicking faces for an individual is indexed by the *y*‐value, and that to anti‐mimicking faces is indexed by the *x*‐value. The circles represent the neurotypical group, and grey squares represent the ASD group.

A significant group difference between ASD and neurotypicals was noted for the (mimicking > anti‐mimicking faces) contrast t‐statistics in the left VS (β_group_ = −0.662, *P* = 0.0265). This group difference was driven by a weaker response in ASD to mimicking faces (mean beta estimate = −0.026) compared to anti‐mimicking faces (mean beta estimate = −0.014), and a higher response in controls for mimicking faces (mean beta estimate = −0.0547) compared to anti‐mimicking faces (mean beta estimate = −0.0592, Fig. 3).

Two control analyses were run, to check if these observed group differences were driven by (i) some participants having detected the pattern of contingencies in the conditioning task, or (ii) ASD participants who had not met the cut‐off on the total ADOS score. Neither of these control analyses significantly altered the pattern of results reported above.

**Figure 4:**
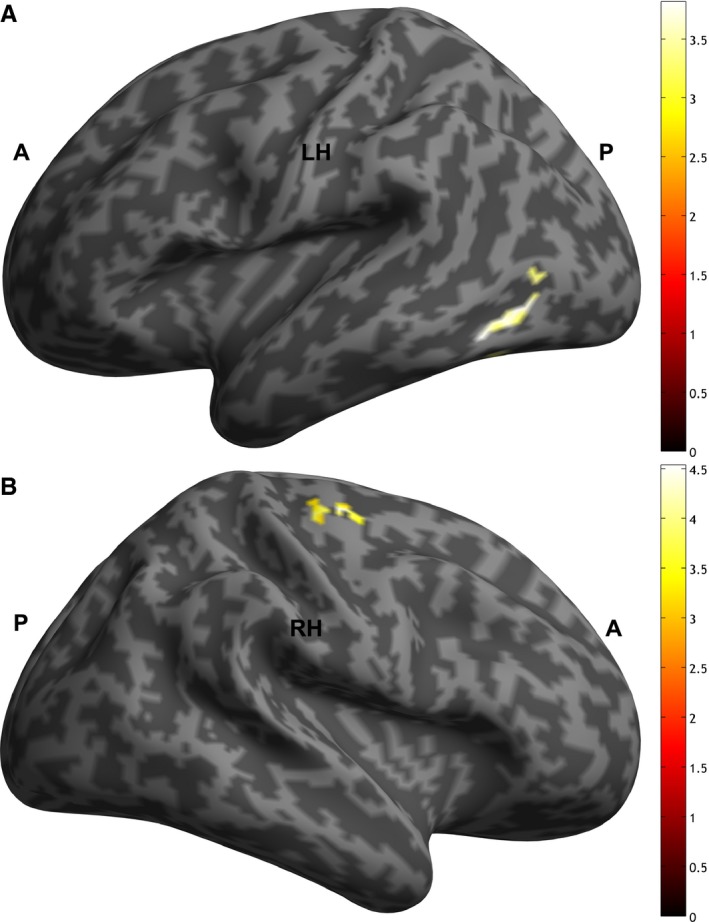
Panel (A) shows the significant cluster of the main effect (mimicking > anti‐mimicking faces) in the left occipito‐temporal junction and fusiform gyrus (MNI −42, −62, −9). Panel (B) shows the significant cluster of the interaction contrast Control (mimicking > anti‐mimicking faces) > ASD (mimicking > anti‐mimicking faces) in the right precentral gyrus (MNI 32, −17, 59).

Whole‐brain analysis for the main effects and the interaction effects are reported for the sake of completeness (Table 2). One cluster in the left occipito‐temporal junction including the fusiform gyrus was significantly more active for the main effect of condition (mimicking > anti‐mimicking faces) (Fig. 4A). One cluster in the right precentral gyrus showed a significant between‐group difference for the contrast (mimicking > anti‐mimicking faces) (Fig. 4B). In other words, the ASD group showed weaker activation in this cluster for mimicking faces vs. anti‐mimicking faces, in relation to the control group.

**Table 2:** Whole‐brain task fMRI results

H	Regions	Cluster size	*P* (FWE)*	*T*	B.A.	*x*,* y*,* z*
Main effect (mimicking > anti‐mimicking faces)						
L	Middle temporal	37	0.24	3.95	37	−42, −62, −9
	Middle occipital			3.81	19	−51, −59, −12
	Fusiform gyrus			3.43	37	−35, −55, −12
Interaction: control [mimicking > anti‐mimicking] > ASD [mimicking > anti‐mimicking]						
R	Precentral gyrus	31	0.048	4.54	6	32, −17, 59

H, hemisphere; L, left; R, right; *P*,* P*‐value; *T*,* T*‐value; B.A., Brodmann's area; *x*,* y*,* z*, MNI coordinates. *Voxel‐level uncorrected *P* < 0.001, cluster‐level FWE‐corrected for the whole brain.

### Trait correlations

**Figure 5:**
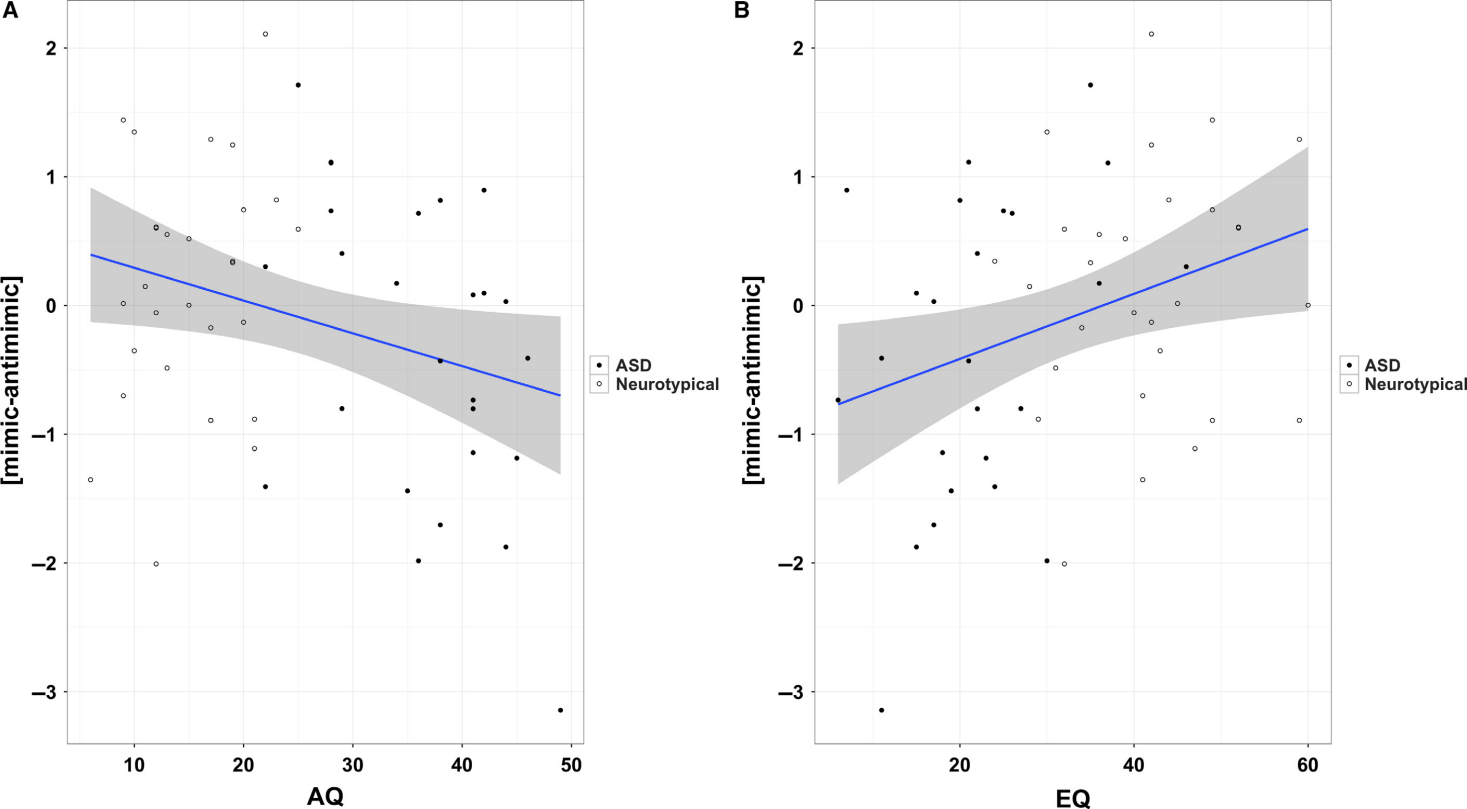
Correlation between autistic and empathy traits and ventral striatal response. Panel (A) shows the negative correlation and the 95% confidence region between AQ and the mean contrast *t*‐values of the left VS in the whole sample (including ASD group and controls). Panel (B) shows the positive correlation between EQ and the mean contrast t‐values of the left VS.

Considering the entire sample irrespective of diagnosis, a significant negative correlation with AQ and a significant positive correlation with EQ with the left VS response contrast (mimicking > anti‐mimicking faces) were found (β_AQ_ = −0.027, *P* = 0.009; β_EQ_ = 0.030, *P* = 0.0023; see Fig. 5A and B). No such relationship was observed in the right VS (β_group_ = 0.066, *P* = 0.809; β_AQ_ = −0.005, *P* = 0.655; β_EQ_ = 0.013, *P* = 0.106). In the whole‐brain analysis, a cluster in the right superior parietal lobule was positively correlated with EQ [centroid coordinates: (32, −78, 46), cluster extent = 35 voxels]. No such significant clusters were noted in the whole brain analysis for the AQ regression. No interaction effect of gender was noted in the regression models [gender × group (β = −0.31, *P* = 0.58), gender × AQ interaction (β = −0.039, *P* = 0.089), gender × EQ interaction (β = 0.034, *P* = 0.108)]. All reported *P*‐values for the correlation analyses are one‐tailed in line with the directional nature of the hypothesized association.

## Discussion

In this study, the VS response to being mimicked in a group of adults with and without ASD was tested using fMRI. The VS comprises neural structures involved in the processing of rewards in humans and other mammals. Individuals with ASD showed a reduced VS response to faces that had mimicked them before compared to those that did not, when compared to controls. Being mimicked has been associated with reward‐related response in the general population in several previous studies as indexed by measures of liking and positive valence (McIntosh, 2006; Kühn *et al*., 2010; Stel *et al*., 2010). In the current study, VS response to being mimicked was negatively correlated with autistic traits (AQ) across the whole sample, as well as within the ASD group, further validating this result and emphasizing the dimensional nature of autism‐related traits across the diagnostic boundary (Robinson *et al*., 2011). In contrast, this VS response correlated positively with trait empathy as assessed with the EQ. Taken together, our observations suggest that reward‐related response to facial mimicry is reduced in individuals with high autistic traits (and low trait empathy), including those with a clinical diagnosis of ASD. These findings are consistent with our previous study in neurotypicals using an almost identical conditioning paradigm, where we tested the effect of mimicry on preferential looking towards the same faces (Neufeld & Chakrabarti, 2016). Greater preferential looking was associated with mimicking compared to anti‐mimicking faces and this effect was positively correlated with EQ, suggesting that empathy was associated with the sensitivity to the mimicry manipulation. As individuals with ASD typically score significantly lower in trait measures of empathy (Baron‐Cohen & Wheelwright, 2004), it is reasonable to assume that the lower reward‐related response to being mimicked in the ASD group in the current study is linked to the lower self‐reported trait empathy in this group. Reduced reward response to social signals has been suggested as a key mechanism involved in the aetiology of ASD in social motivation‐based accounts of autism (Schilbach *et al*., 2010; Chevallier *et al*., 2012).

Crucially, instead of testing the immediate impact of being mimicked, this study investigated a conditioned reward learning effect after being mimicked by an individual repeatedly. In the neurotypical group, this manipulation led to mimicking faces to be associated with higher likeability ratings than anti‐mimicking faces, and a similar trend was noted for the ventral striatal response. These results are in line with other studies that have demonstrated lasting benefits for those who mimic, such as being liked more (Chartrand & Bargh, 1999) or earning more money (van Baaren *et al*., 2003). Mimicry has been suggested to play a key role in the formation of human social bonds from early infancy. Parents show frequent mimicking behaviour with their babies, to entertain them, to attract their attention to something in the environment and to further enhance mutual rapport. This effect has been investigated in previous studies showing that babies look and smile longer at adults who are mimicking them compared to adults mimicking another baby or performing only temporally but not structurally congruent movements (Meltzoff & Brooks, 2001). It is possible that a reduced reward‐related response to being mimicked in ASD might underlie some of the difficulties in social learning and formation of social bonds in these individuals. However, future longitudinal studies should test this hypothesis in infants to directly test this claim.

This set of results explores the link from mimicry to reward, and how it is affected by autism‐related traits. It complements the evidence suggesting weak links from reward to mimicry in people with high autism‐related traits, as demonstrated by previous studies. These previous studies had shown reduced spontaneous facial mimicry and frontostriatal connectivity in individuals with high autistic traits, when presented with rewarding social stimuli (happy faces; Sims *et al*., 2012, 2014).

Three caveats need to be considered while evaluating the evidence presented above. First, the group difference in the VS response (i.e. the group × mimicry interaction effect) was driven by a higher VS response to mimicking vs. anti‐mimicking faces in the neurotypicals, as well as an opposite pattern in the ASD individuals. The trend for higher VS response to mimicking vs. anti‐mimicking faces in neurotypicals is in the expected direction and is supported by previous studies mentioned above. However, the higher VS response to anti‐mimicking faces in ASD was unexpected. While reward response to anti‐mimicking stimuli needs to be investigated in a separate study, we speculate that the pattern observed in the current study could be driven by potentially higher salience for non‐matching actions in ASD (i.e. anti‐mimicking faces). While both mimicking and anti‐mimicking faces performed expressions contingent to those of the participant, ASD individuals might have found the anti‐mimicking face to be more enjoyable due to their performing an opposite/unexpected expression. Future studies should systematically explore the response to anti‐mimicking stimuli in ASD.

Second, the significant group effect on the differential VS activity for mimicking vs. anti‐mimicking faces was found only in the left hemisphere in the current study. Similar left lateralization of striatal activity has been found in response to monetary and social reward in neurotypicals (Koepp *et al*., 1998; Delgado *et al*., 2000; Izuma *et al*., 2008), as well as in pathological gamblers (Steeves *et al*., 2009; Linnet *et al*., 2010). Another potential source of this left lateralized result could be due to the left lateralized frontostriatal dysfunction previously noted in autism (Rinehart *et al*., 2002) and is consistent with the suggested left hemisphere dysfunction in autism (Floris *et al*., 2013).

Finally, the conditioning phase of the current paradigm involved the stimuli faces making both happy and sad expressions. While this task enabled an exploration of the impact of high vs. low mimicry *per se* irrespective of stimulus valence, it did not allow for parsing the reward‐related neural response to a mimicking happy vs. a mimicking sad face. Accordingly, an interesting direction for future research would be to explore whether the rewarding effect of mimicry is modulated by the valence of the emotion expressed by the mimicking/anti‐mimicking faces.

An exploratory analysis at the whole‐brain level revealed a main effect of mimicking vs. anti‐mimicking faces in the left occipito‐temporal cortex and fusiform gyrus, regions in which the activity has been associated with visual attention specific to face processing (Wojciulik *et al*., 1998; Reddy *et al*., 2007). It is in line with our previous eye‐tracking study showing that participants showed gaze bias towards mimicking faces (Neufeld & Chakrabarti, 2016). A significant interaction effect was also in the right prefrontal cortex, in which the neurotypical group showed stronger activation difference for mimicking vs. anti‐mimicking faces than the ASD group. The significant cluster is located around the junction of hand and face area within the motor/premotor cortex, which is known to be active during deliberate mimicry of facial expressions (Lee *et al*., 2006). This result suggests that neurotypical participants have potentially greater preparatory motor activity in response to a face that has previously mimicked them more, similar to previous results using EEG (Hogeveen *et al*., 2015).

This study, as several other previous studies in a recent systematic review, lends further evidence to the reward‐related/positive response to being mimicked (Hale & Hamilton, 2016). It opens up broader theoretical questions on why should being mimicked lead to a reward‐related response. Contingency alone cannot account for the reward‐related response, as both the mimicking and the anti‐mimicking response are equally contingent. Recognition of self‐other overlap (‘like‐me’) can be a potential explanation (Meltzoff & Brooks, 2001), although it leads to a further questions on why the self should be associated with reward response. It should further be acknowledged that the rewarding effect of being mimicked might be different when detecting the mimicry of one's true emotional expression (due to experienced emotion) than that of a posed emotion expression. Future research should hence test these observed effects within a more naturalistic setting.

In conclusion, we observed reduced reward‐related response in the VS to facial mimicry in ASD individuals in comparison with neurotypicals. It suggests that in adults with high autistic traits including those with a clinical diagnosis of ASD, being mimicked is not perceived to be equally rewarding. This observation offers a potential clue to understanding the reason why some individuals with ASD find it so difficult to build social rapport. Future studies should test the validity of these results in a sample of children with ASD.

## Conflict of interests

The authors declare no potential source of conflict of interests.

## Author contributions

B. Chakrabarti developed the study concept. B. Chakrabarti and J. Neufeld contributed to the study design. Data collection was done by J. Neufeld. C.T‐Hsu & J.Neufeld contributed to the analysis. Draft of manuscript was written by C.T.‐Hsu, while both other authors contributed to critical editing of the draft. All authors approved the final version of the manuscript for submission.

## Data accessibility

All data are available upon request from the corresponding author.

**Abbreviations**
AQ: Autism quotient
ASD: Autism spectrum disorders
BOLD: blood oxygen level dependent
EEG: electroencephalography
EMG: electromyography
fMRI: functional magnetic resonance imaging
FWE: family‐wise error
ICA: independent component analysis
IFG: inferior frontal gyrus
MNI: Montreal Neurological Institute
ROI: region of interest
VS: ventral striatum
